# Supplementary material for: Engineered Cross-Linked Silane with Urea Polymer Thin Durable Coatings onto Polymeric Films for Controlled Antiviral Release of Activated Chlorine and Essential Oils
Source: J Funct Biomater. 2023 May 12;14(5):270. doi: 10.3390/jfb14050270 (PMC10218995; doi:10.3390/jfb14050270)
Supplement: Supplementary file 1 [file jfb-14-00270-s001.zip › jfb-2303926-supplementary.pdf]

SUPPORTING INFORMATION

Engineered Cross-Linked Silane with Urea Polymer Thin Durable Coating onto Polymeric Films for Controlled Antiviral Release of Activated Chlorine and Essential Oils

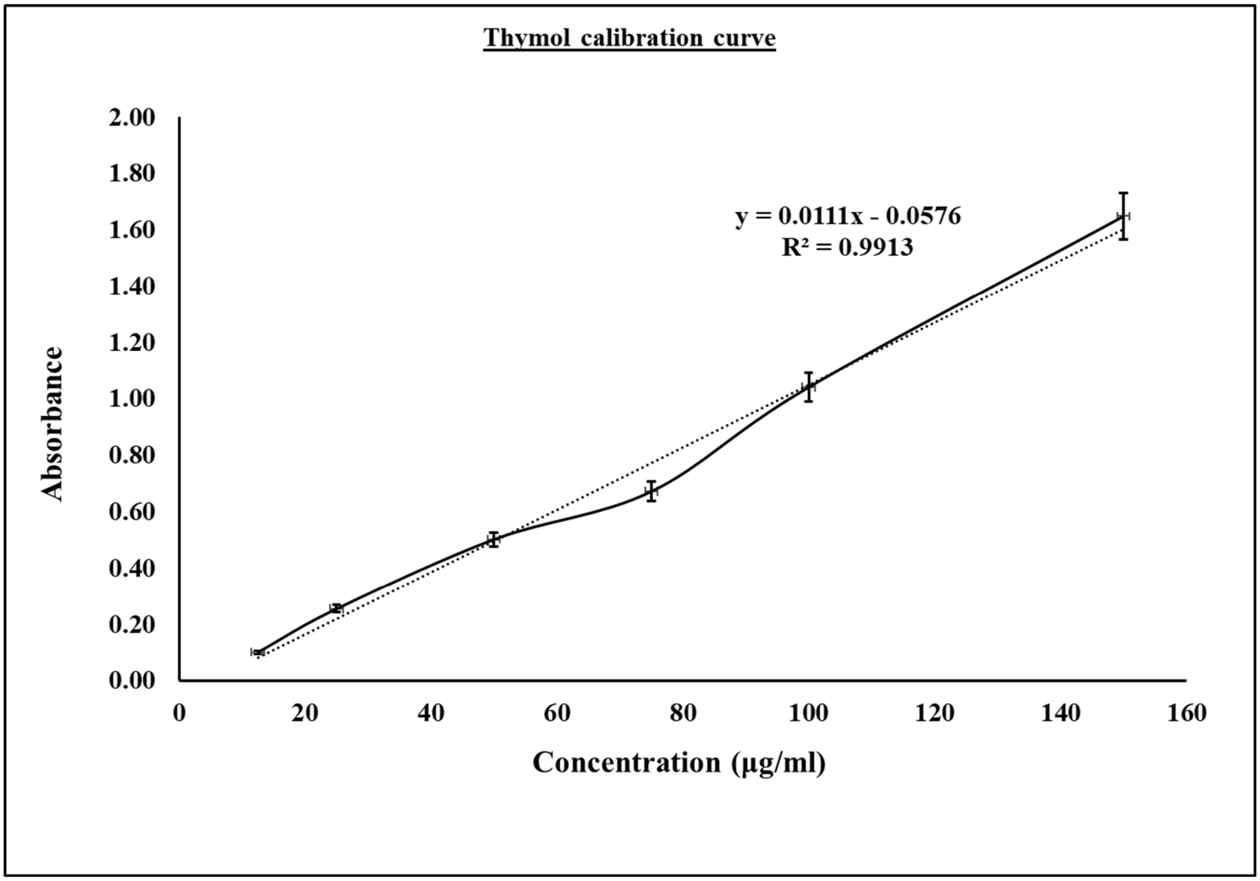

Figure S1: Thymol calibration curve.

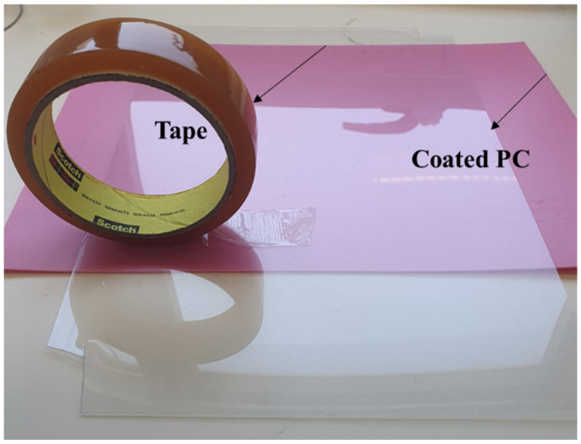

Figure S2: Adhesive test of coated film by tape. The tape was firmly pressed onto the PC/SiO_2-urea film and slowly peeled off. After 25 repetitions, the ATR test was applied to the film.

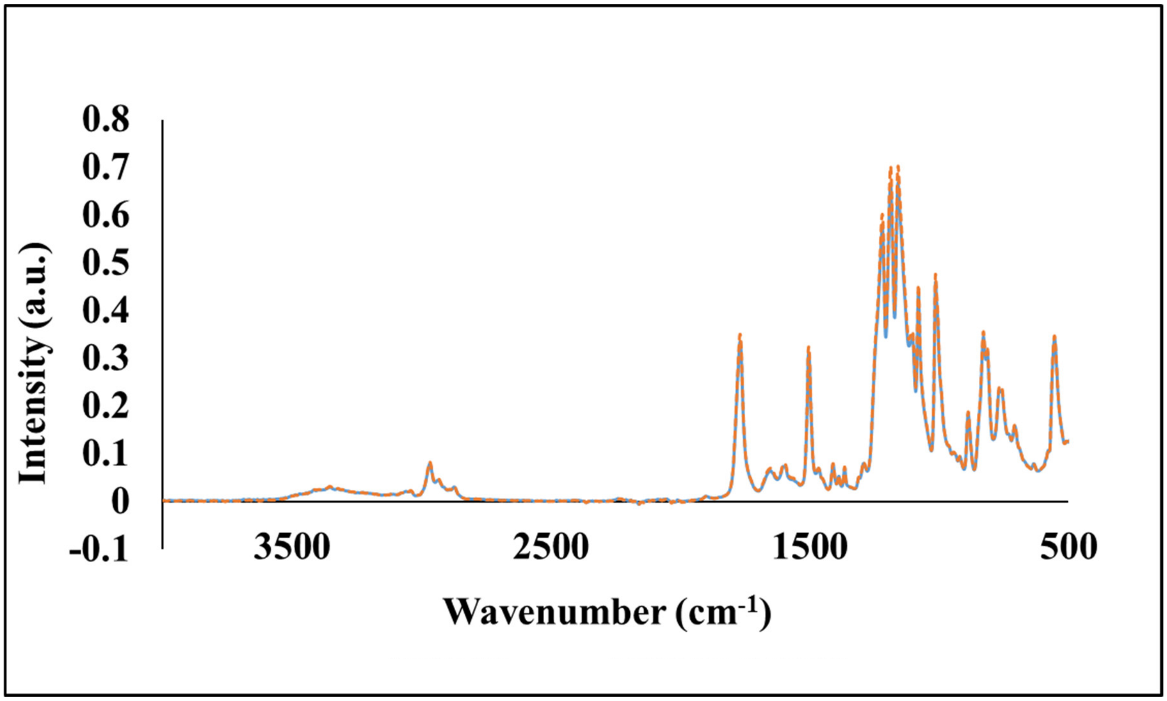

Figure S3: FTIR spectra of PC/SiO_2-urea before (blue) and after (orange) tape test.

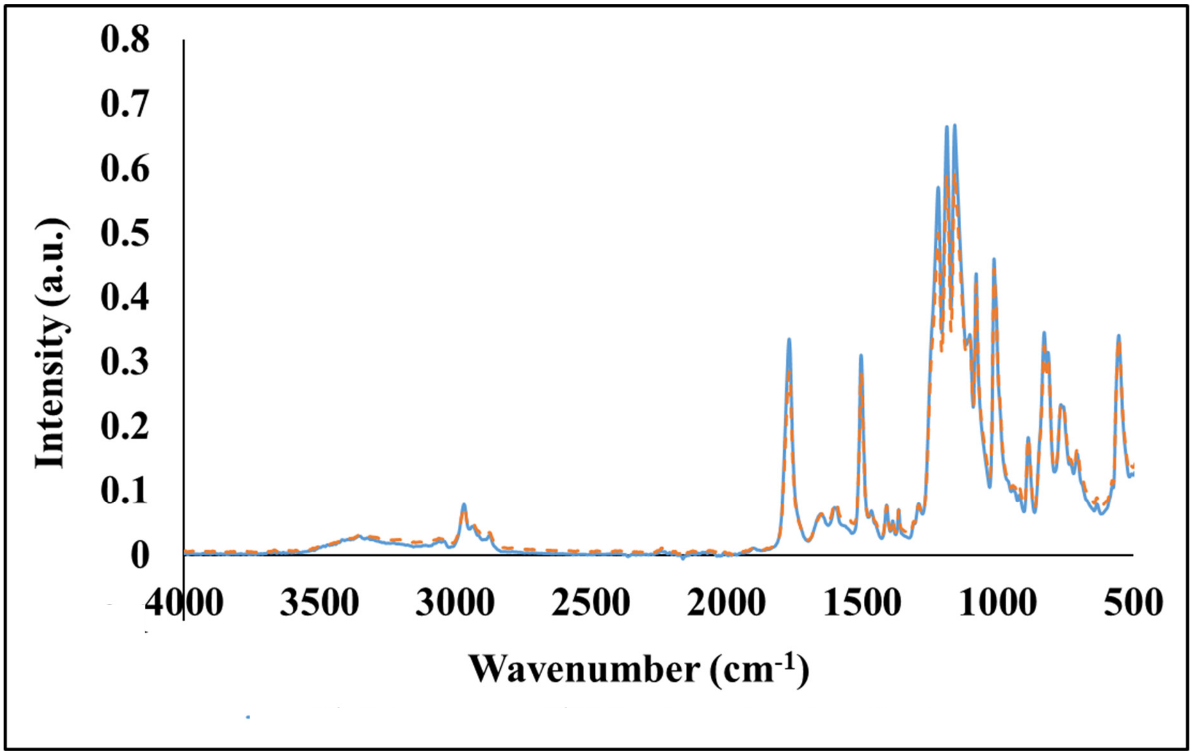

Figure S4: FTIR spectra of PC/SiO_2-urea before (blue) and after (orange) 6-month soaking test in water.
